# Comparison of the liver function and hepatic specific genes expression in cultured mesenchymal stem cells and hepatocytes

**Published:** 2014-01

**Authors:** Zahra Nikoozad, Mohammad Taghi Ghorbanian, Arezou Rezaei

**Affiliations:** 1 School of Biology, Damghan University, Damghan, Iran; 2 Institute of Biological Sciences, Damghan University, Damghan, Iran

**Keywords:** Albumin, Glycogen, Hepatocytes, Liver genes, Mesenchymal stem cells

## Abstract

***Objective(s):*** Stem cell therapy is believed to be as a promising treatment strategy for tissue repair and regeneration. The plasticity specification of the adult stem cells, such as MSCs, has enabled that these cells to be used in the treatment of a broad spectrum of diseases like liver disorders. In this study, the production of urea and Albumin (Alb), glycogen storage, and expression of some liver genes including α-fetoprotein (AFP), Alb, cytokeratin18 (CK18) and cytokeratin19 (CK19) was compared between mesenchymal stem cells (MSCs) and isolated rat hepatocytes.

***Materials and Methods:*** The MSCs were isolated from rat femurs and tibias and cultured in α-MEM, DMEM and RPMI mediums supplemented with serum. Hepatocytes were isolated from Rat livers and cultured in DMEM with serum. The expression of AFP, Alb, CK18, and CK19 genes was evaluated using the reverse transcription-polymerase chain reaction (RT-PCR). Furthermore, the synthesis of albumin and urea of the cells was measured.

***Results:*** *In vitro* conditions, MSCs and hepatocytes exhibited the characteristic functions of the liver such as capacity to synthesize Alb, urea, the storage of glycogen. In this study, the expression of some liver genes such as AFP, Alb, CK18 and CK19 at mRNA levels was also shown.

***Conclusion:*** The results showed that MSCs exhibited some liver functions, and may be considered as an alternative source for adult stem cell transplantation in liver repair due to the excellent proliferation and differentiation capacities.

## Introduction

The increase of liver failure as a high risk disease, shortage of liver donors and the problems associated with liver transplantation have made researchers suggest stem cell-based therapies as a supplemen-tary treatment to stem cell transplantation for this disease (1). Stem cells are known by their origin tissues, which include embryonic stem cells (ESCs) and adult stem cells. “Oval cells” are one population of adult stem cells that resides in terminal branches of the intrahepatic biliary tree. In addition to oval cells, stem cells in other organs such as mesenchymal stem cells (MSCs) and hematopoietic stem cells (HSCs) of bone marrow play a pivotal role in regeneration (2, 3). In reality, the application of stem cells derived from different sources and their differ-entiation to liver cells in laboratory or application of non-induced cells with the normal condition have been the most important cell sources in the regene-rative medicine (4-7). A subpopulation of MSC isolated from bone marrow of human, mouse and rat, express hepatic markers such as CK19, α-fetoprotein, albumin and CK18 (8). MSCs showed differentiation potentials into hepatocytes when co-cultured with injured liver cells (8, 9). This procedure suggests that bone marrow cells contribute to the normal renewal and regeneration of the liver tissue. *In vivo* condi-tions, interactions between adjacent parenchymal and non-parenchymal cells in the liver regeneration process cause the regulation and modulation of growth, migration and differentiation of the hepato-cyte progenitor cells. In fact, created regulatory factors and molecular signals of cytokines induce the MSCs differentiate to the liver cells, depending on their microenvironment (10). Regenerative medicine employs stem cells to overcome the shortage of donors, operative damage, and organ rejection (8). MSCs possess the abilities of hepatic engraftment, and in addition, their easy accessibility and quick *in vitro* expansion make MSC an ideal resource for the clinical use (11). In this research differentiation potential of MSCs into hepatocytes in α-MEM, RPMI and DMEM mediums was studied to see the influence of culture media on cell growth and differentiation. Hepatic-differentiated cells are characterized by the expression of hepatocyte specific genes (8). Accord-ingly, in this study some characteristic functions of liver cells including the capacity to synthesize albumin, urea, and the storage of glycogen were reported. Moreover, the expressions of some hepatocyte specific genes such as AFP, Alb, CK18 and CK19 in the MSCs and hepatocytes were investigated.

## Materials and Methods

***Animals***

Experiments were performed in accordance with the recommendations and approval of the Ethics Committee on Animal Experiments of Damghan University. Male Wistar rats, aged 6-8 weeks were used. The animals were housed in stainless steel cages with free access to standard chow and water and exposed to 12 hr light-dark cycles under standard animal laboratory conditions at room temperature. 

***Isolation and culture of MSCs***

The animals were sacrificed using chloroform anesthesia. Rat MSCs were obtained from the bone marrow of femurs and tibias using the method of Azizi *et al* (12). The cells were cultured in Alfa- Dulbecco’s minimal essential medium (α-MEM), Dulbecco’s modified Eagle’s medium (DMEM) and RPMI 1640 (Gibco) supplemented with 10% fetal bovine serum (FBS) (Gibco), 1% penicillin and streptomycin (Gibco). The cells were seeded in 25 cm^2^ tissue culture flasks) Falcon) and incubated at 37°C and 5% CO_2_. After 48 hr non-adherent cells were removed by replacing the medium. After the cultures reached the optimum confluence, cells were lifted by incubation with 0.25% trypsin and 0.02% EDTA (Merck) at 37°C for 3–4 min and the subculture of the cells were re-plated at a density of 4×10^5^ cells/cm^2^_. _Cell density (cell count) and cell viability were determined using a Neubauer hemocytometer as explained by Davis JM (13) and MTT assay (14).

***Isolation and culture of hepatocytes***

After a complete anesthesia, liver tissue samples were harvested and rinsed with phosphate-buffered saline (PBS). Sections were then placed in microtubes (1.5 ml) with collagenase solution (0.2%) and incubated for 40 min at 37°C. Resulting cell clumps were dissociated by gentle pipetting and the effect of the collagenase solution was neutralized with medium supplemented by the serum (1 ml). The cell suspension was filtered through a 200 μM pore size nylon mesh and centrifuged at 400 g for 5 min. The supernatant was removed and the cell pellets were resuspended in DMEM D-glucose (1 ml) supplemented with 10% fetal bovine serum (FBS) and placed in falcon tissue culture dishes.

***MTT [3-(4,5-dimethylthiazol-2-yl)-2,5-diphenyl-2H-tetrazolium bromide] assay***

Cell viability and proliferation rate were detected by MTT assay (14). Briefly, 2×10^4^ cells at passage 3 or 4 per well were seeded in 96-well plates, and incubated in 200 µl α-MEM supplemented with 10% FBS. After 48 hr, the supernatant was discarded and replaced with 100 µl medium supplemented with 10 µl MTT solutions (5 mg/ml, Sigma) and incubated for 4 hr at room temperature. Then, 85 µl of supernatant was discarded, afterward 50 µl DMSO added and incubated for 10 min, after different time intervals, absorbance value was measured in ELISA reader (Biotek) at 540 nm. The experiment was repeated over 5 times for each group.

***Immunocytochemistry***

After 3 or 4 pssages immunocytochemical testing was performed to confirm the MSC lineage. Briefly, the cells cultured at a density of 5×10^4^ cells/cm^2^ on gelatin coated cover slips in culture dishes were exposed to (1:20) mouse monoclonal anti-human CD71, (Sigma, C2063) and (1:20) CD90 antibodies mouse (Miltenyi Biotec, 130094524). Samples were fixed with 4% paraformaldehyde for 20 minutes. The cells were then treated with 0.3% Triton X-100 for 15 min and 10% goat serum at room temperature for 15 min. Next, the cells were incubated overnight at 4°C and humid conditions to the primary antibodies included CD71 and CD90. FITC conjugated anti-rat secondary antibody (1:100) (Sigma, F6258) was then added and the cells were incubated in the dark. The slides were examined by florescence microscopy (Nikon Eclipse-E600) and the images of cells were taken with a digital camera (Nikon Digital Camera, 1200 DXM). The positive cells for each marker were counted under the fluore-scence microscope and expressed as a percentage of the total cell number (stained and non-stained cells).

**Table 1:** The primers used for reverse transcription-polymerase chain reaction (RT-PCR) analysis

Gene	Size (base pairs)	Primer sequence	Accession number
β_2_M	318 bp	F: 5'-CCg TgA TCT TTC Tgg TgC TT-3' R: 5'-TTT Tgg gCT TCA gAg Tg-3'	NM-012512
AFP	400 bp	F: 5-CCT-gAC- Agg-gAA-gAT- ggT-gAg-<C>-3׳ R:5׳ gCA-CTT-CTC-CAA-gAg-gCC-AgA-<g>-3׳	NM-012493
Alb	400 bp	F: 5׳-AAA-CgC-CgT-TCT-ggT-TCg-ATA-<C>-3׳ R:5 ׳- ggg-CTT-gTg-TTT-CAC-CAg-CT-<C>-3׳	NM-134326
CK18	401 bp	F: 5 ׳- TgA-ggg-CTC-AgA-TCT-TTg-C-<g>-3׳ R:5׳-CTT-gTC-CAg-TTC-CTC-ACg-gTT-<C>-3׳	NM-053976
CK19	445 bp	F: 5'- ACT-TgC-gCg-ACA-ACA-TCC-TT-<g>-3' R: 5'-ACA-gCg-ACC-Tgg-gTg-TTC-AA-<C>-3'	NM-199498

**Figure 1:**
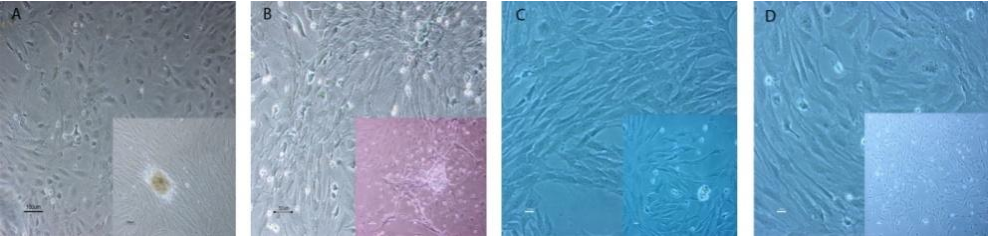
Phase contrast photomicrograph of (passage 3) in MSCs. A: α-MEM, B: RPMI, C: DMEM and D: hepatocytes at (passage 3)

**Figure 2:**
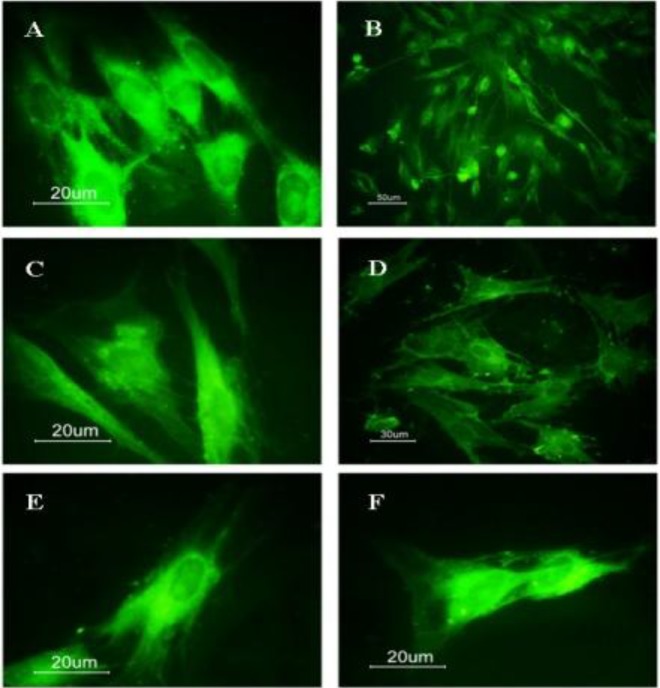
Representative fluorescence photomicrographs. MSCs immunostained with Anti CD71 and Anti CD90 in mediums: α-MEM, A: CD71 B: CD90. RPMI, C: CD71 D: CD90. DMEM، E: CD71, F: CD90. Positive reaction of MSCs for CD71 and CD90 markers

***RT-PCR***

Total RNA was extracted from the cultured cells (3 or 4^th^ passages) and rat liver tissue as the positive control using the Cinnagen- kit. Standard reverse transcription reaction was performed using 0.5µg total RNA according to instructions provided by the cDNA synthesis Kit (Fermant-k1622 kit). The PCR mixture (25 μL total volume) consisted of 1 μl of cDNA-template, 0.5 μl of each primer (10 picoMol), 2.5 μl of 10 × PCR-buffer, 0.8 μl of 10 mMol dNTP´s, 0.8 μl of 50 mMol MgCl_2_ and 0.25 μl of polymerase (Ampli-Taq, Cinnagen). The sequence of primers, size and accession number of fragments in the gene bank are listed in Table 1. The PCR was carried out in a thermal cycler (Eppendorf, Germany) with the following cycle profile: initial denaturation at 94°C for 2 min, and 34 cycles of 94°C for 30 sec, annealing at 64°C for 30 sec, elongation at 72°C for 30 sec, and final extension at 72°C for 5 min. Gene expression was assessed in different cycles. No band was observed below 25 cycles. All reactions were performed in triplicate and controlled by negative RT (no enzyme) and no-template controls. The expression of genes was determined in rat liver tissue as the positive control. Products of PCR were analyzed with 2% agarose gels and ethidium bromide staining. Fragment sizes were calculated by application of the DNA ladder. All PCR reactions were normalized to β2M as an internal control to measure the genes expression intensity. The expression intensity of the genes was measured using the UVIdoc software (densiometer) and compared with β2M. 

***Biochemical analysis***

Urea concentration and albumin level were determined using commercial kits (parsazmoon) in three culture medium supernatants of MSCs and hepatocytes. Periodic acid–Schiff (PAS) staining technique was used for the demonstration of glycogen storage (15). All assays were performed 5-7 times for each condition.

***Statistical analysis***

The comparison between diverse groups was performed by one-way analysis of variance (ANOVA) with Tukey's and LSD complementary test employing “SPSS” software (version 16) with a significance level set at *P*≤ 0.05. The resultant data are presented as the mean standard deviation (SD).

## Results

In the first days of culture, the MSCs grew as a colony-forming unit before reaching to the confluence and subculture of cells. These clonal cells aggregation represented as a spherical structure were observed before reaching to high confluence (Figure 1). The MSCs showed three morphologies in cell culture. The colonies contain small spherical shape cells that rapidly develop a greater number of cell populations in primary cultures. One group of the cells had a spindle shape or fibroblast-like morphology (16) and the other group exhibited a large broadened cuboidal shape (Figure 1). The comparison between culture medium showed that the MSCs had the best conditions based on the cell morphology, growth and the replication rate in α-MEM medium. The MSCs in the α-MEM medium showed a high growth speed measured by hemocytometer method and MTT assay (data not shown). Apart from the α-MEM, among the three mediums, the MSCs had improved conditions in the RPMI medium compared with the DMEM medium. After 1 or 2 cellular passages, the spindle and fibroblast-like morphology of the cultured cells changed into the flat shape in the DMEM medium. In the DMEM medium, the passage of cells took a longer time in comparison to others. In the higher passages, the cells were observed with a broad and cuboidal form and short processes in three mediums. 

**Figure 3:**
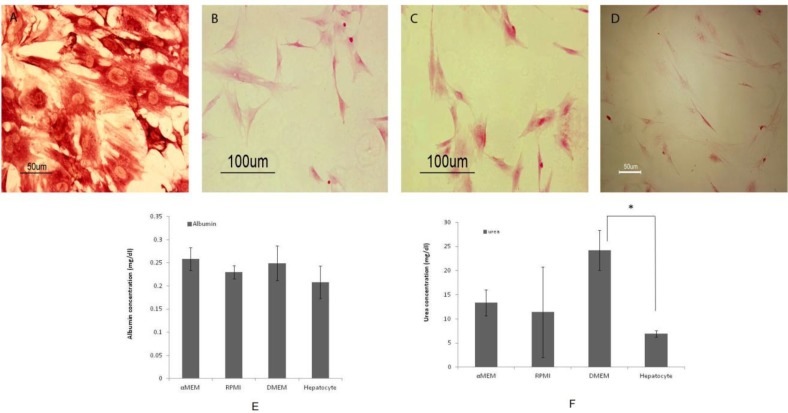
Glycogen storage determined by periodic acid-Schiff (PAS) staining of MSCs in A: α-MEM, B: RPMI, C: DMEM, D: Hepatocytes with highest reaction. (E). Albumin production in the experimental groups. Measurement of the amount of albumin secretion in the supernatants did not have statistically significant differences between various conditions. (F). Urea production in the experimental groups. The assessment of urea concentrations showed a significant difference between hepatocytes and MSCs in DMEM medium (*P*≤0.05)

During the first days of culture, hepatocytes exhibited the colonial growth and aggregation, forming large clusters such as the MSCs primary culture. Hepatocytes grew slowly as compared to the MSCs and were observed in a big size with one or two central light large round nucleus and prominent nucleolus (Figure 1A). Liver hepatocytes progressed to the 4^th ^passages in culture. 

The positive reaction of MSCs for CD71 and CD90 markers were compared in three mediums. The resultant data are expressed as means ± standard error of the mean such as follows: Immunocyto-chemistry staining using antibody to CD71 in the α-MEM medium: (92/2 ± 1/39), in the DMEM medium: (91/4 ± 1/16), in the RPMI medium: (92 ± 1/48), also for the CD90 marker: (94/2 ± 0/8), (92/4 ± 0/81), (93/6 ± 0/74) respectively (Figure 2). The results showed that the cells extracted from the bone marrow can express the surface markers of the MSCs.

The glycogen storage was observed in mesenchymal and liver cells by the periodic acid–Schiff staining (PAS). The comparison between culture medium showed that the hepatocytes had the highest reaction with PAS (Figure 3). The measure-ment of albumin secretion in the supernatants did not have statistically significant differences under various conditions (Figure 3E). The assessment of urea concentrations showed that there was signify-cant difference between hepatocytes and the MSCs in the DMEM medium (Figure 3F). 

**Figure 4:**
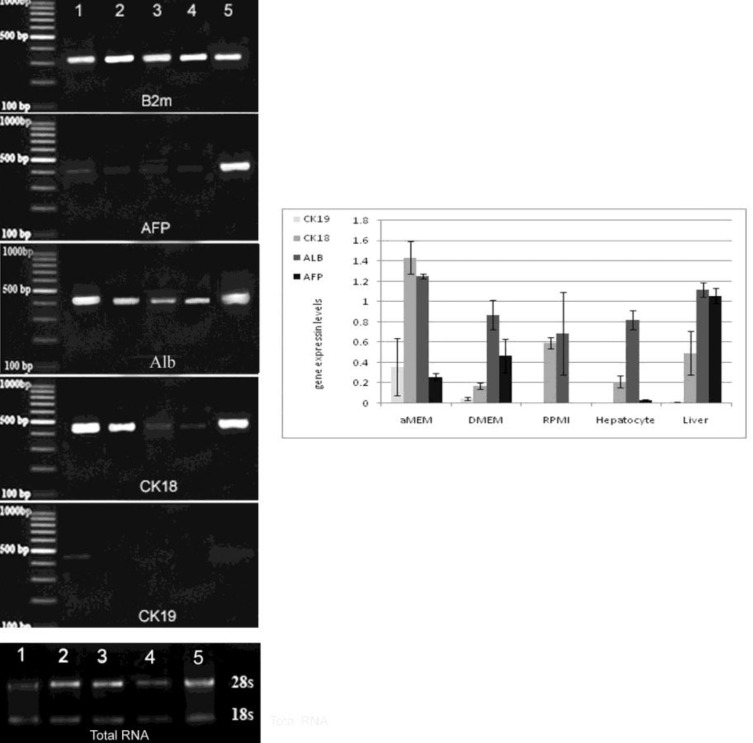
RT-PCR results showed that MSCs and hepatocytes could express a number of hepatocyte-specific genes in different medium. Lane 1α-MEM, lane 2: RPMI, lane 3: DMEM, lan 4: hepatocytes and lane 5: liver. (below) Electrophoresis of total RNA which showed of MSCs in (1): α-MEM, (2): RPMI, (3): DMEM, (4): Hepatocytes and (5): liver. Liver tissue was used as positive control. (Graf) The comparison of semi-quantitative expression of the genes in different cell culture conditions. The comparison of gene expression in different groups (apart positive control) showed no significant difference in terms of AFP and Alb (*P*≤0.05). Gene expression density of CK18 showed that, there were significant differences between MSCs cultured in α-MEM and the DMEM & hepatocyte, so between MSCs cultured RPMI medium and hepatocyte (*P*≤0.05)

The quantitative and qualitative analysis of RNA in all groups indicated a normal RNA with healthy 18S and 28S bands (Figure 4). The PCR products of the MSCs in the α-MEM, DMEM, RPMI mediums, hepatocytes and liver tissue (as the positive control) were resolved by electrophoresis. The resultant data revealed the expression of β_2_M (Housekeeping gene), AFP, Alb, CK18 in all groups and low expression of CK19 in the BMSCs with the α-MEM medium and in the liver tissue, while the expression of CK19 was not detected in other groups. 

## Discussion

The aim of the present study was to investigate the expression of some specific liver genes in MSCs without application of any inducers or coculture system. Three different mediums were used to identify the best culture medium for the occurrence of some functional markers of liver. 

In addition to the regenerative role of liver cells, there is cooperation between liver stem cells and BMSCs (Extra hepatic stem cells). The MSCs play a major role in both inflammation and remodeling of tissues. Following a liver injury, cells from the bone marrow are released into the circulation, migrate to the liver and differentiate into hepatocytes (3, 17, 18). The HSCs and MSCs are the most important candidates and possess the unique capacity for liver regeneration. The BMSCs contributes to the regeneration of parenchymal and non-parenchymal liver (hepatic) cells and are capable of being differentiated into hepatocytes (2). This research has demonstrated that the MSCs can be developed into hepatocytes following in vivo transplantation (19). However, under *in vitro* conditions, these cells may differentiate to functional hepatocytes in the presence of growth factors such as the hepatocyte growth factor (HGF) and FGF-4 (20-22). These functional hepatocytes under *in vitro* conditions also possess functional characteristics of hepatocytes, such as secreting urea and albumin, taking up LDL and storing glycogen (23). Finally, the differentiation of MSCs into particular cell lineages under appro-priate culture conditions makes these cells being capable in the treatment of many diseases (19).

 In this research, apart from the study undertaken on the characteristics of the MSCs in three culture mediums, the proliferation ability, the expression of hepatocyte specific genes and the function of these cells were compared with hepatocytes culture. The MSCs in the α-MEM medium indicated a high viability and replication capability in a way that after 6 to 7 days culture reached 80-90% confluence. Doubling times and the growth curve could represent differences of growth and proliferation (replication) in three mediums considered. The growth speed of hepatocytes was much slower than that of MSCs and so that they maintained the proliferation capacity no longer to 4^th ^passage. 

To identify mesenchymal cells, the immunocyto-chemical testing was performed using specific markers CD71 and CD90. The immunocytochemical identification of cultured MSCs in three mediums for cell surface antigen markers CD71 (24-26) and CD90 (14, 24, 27-29) showed that these cells represented a similar and positive reaction to markers. 

The analysis of the liver genes expression in the MSCs, hepatocytes and liver tissue at the mRNA level showed that AFP, Alb and CK18 have expressed in all groups. Additionally, CK19 had a low expression in liver tissue and the MSCs in α-MEM, and not detected in other groups. Since the hepatocytes are the main cell types resident in the liver, RT-PCR results at mRNA level showed that Alb (typical marker of mature hepatocytes) and CK18 (marker of hepatocytes and bile duct cells) expressed at high levels. In addition, AFP appeared to be expressed on average and also CK19 (marker of bile duct cells) expressed at very low levels in liver tissue. Useful stem cell-derived hepatocytes need to express not only the genes found in mature liver cells, but also the level of the expression need to be at or near those found in the normal liver (30). 

The MSCs were co-cultured on normal or CCL4-injured liver tissue. After 10 days of co-cultures of

the MSCs with either normal or injured liver tissues, the RT-PCR analysis of hepatocyte-like phenotypes revealed the expression of both the AFP and albumin (31). Wang *et al *(2004) demonstrated that after 4 days of cultures in the presence of growth factors such as HGF, EGF, aFGF and bFGF, the BMSCs showed a remarkable transition from the fibroblast-like morphology to round and epithelial cells. The RT-PCR analysis showed that before differentiation, mesenchymal cells expressed CK18 and differenti-ated cells exhibited significantly higher levels of expression of albumin and CK18 (32). 

Our results showed that the MSCs cultured in the α-MEM medium had the same gene expression profile which was similar to the adult liver. In contrast, the gene expression profiles of liver genes in the MSCs cultured in the RPMI and DMEM mediums were similar to the gene expression profiles of hepatocytes. High expression levels of specific hepatocytes genes such as Alb and CK18 and lack of CK19 expression demonstrated that the two mediums of RPMI and DMEM had the potential to support or induce the MSCs into a hepatocyte-like cell. 

The kind and rate of expression of liver specific genes during liver development can be used to evaluate the differentiation capacity of stem cells for transplantation (33). In this study, the comparison of a semi quantitative expression of the genes in different cell culture conditions revealed the differ-entiation capacity of these cells. Various studies reported the expression of hepatocytic markers (Alb, Ck8 and CK18), bile duct markers (CK-7, CK-19 OV-6 and A6), hepatoblast marker (AFP) and hematopo-ietic stem cell (HSC) genes (such as Thy- 1, Sca-1 and c –kit) in oval cells (3, 34- 36). In summary, the results of the present study show that the MSCs have the capability of expressing liver and hepatocytes specific genes in culture. Nevertheless, the liver is responsible for a variety of biochemical functions, including the metabolism of amino acids, lipids and carbohydrates, the detoxification of xenobiotics and glyconeogenesis, the synthesis of cholesterol, bile and phospholipids. In additions liver function includes the synthesis of serum proteins such as albumin, fibrinogen, α- globulin and β- globulin and coagulation factors (37). The evaluation of albumin in culture supernatants of MSCs reveals that there is no significant difference between MSCs and hepatocytes. The assessment of urea concentrations in experimental groups shows that there is significantly difference between hepatocytes and MSCs in the DMEM medium despite the fact that there is no significant difference between hepato-cytes and MSCs in other two mediums. The amount of urea in cultured hepatocytes was lower than the other experimental groups, despite the fact that this activity is one of the most important functions of hepatocytes. Since the maintenance of the hepato-cytes function is difficult in culture, it seems that this difference may be due to slow cell growth and difference in cell culture conditions (32, 38). It is assumed that the bone marrow stem cells contribute in liver regeneration because the results show that the MSCs and hepatocytes, in addition to the synthesis of albumin and urea, express some of liver genes such as AFP, Alb, and CK18 at the mRNA level. Hence cell therapies can be represented as one of the most promising alternative techniques to liver transplantation (2, 39). As the isolation of human hepatocytes is difficult and inefficient livers would still be required as a source of cells. Furthermore, the hepatocytes cannot be effectively expanded in culture (40, 41). Therefore, studies have concentr-ated on investigating the capacity of various type of stem cells that can be readily isolated using noninvasive procedures, to give rise to the hepato-cytes both *in vitro* and *in vivo* (5). 

## Conclusion

According to our data growth speed of MSCs was higher than that of hepatocytes. In addition, the MSCs were similar to the hepatocytes in some liver func-tion parameters and expression of some hepatic genes in culture media without any supplemented growth factors. In conclusion, further work needs to be done to establish whether MSCs can be an ideal candidate in cell culture, animal models and clinical trials for cell transplantation in the liver regenera-tion. 

## References

[1] Kallis YN, Alison MR, Forbes SJ (2007). Bone marrow stem cells and liver disease. Gut.

[2] Oertel M, Shafritz DA (2008). Stem cells, cell transplantation and liver repopulation. Biochim Biophys Acta.

[3] Cantz T, Manns MP, Ott M (2008). Stem cells in liver regeneration and therapy. Cell Tissue Res.

[4] Seki T, Yokoyama Y, Nagasaki H, Kokuryo T, Nagino M (2012). Adipose tissue-derived mesenchymal stem cell transplantation promotes hepatic regeneration after hepaticischemia-reperfusion and subsequent hepatectomy in rats. J Surg Res.

[5] Almeida-Porada G, Zanjani ED, Porada CD (2010). Bone marrow stem cells and liver regeneration. Exp Hematol.

[6] Fürst G, Schulte am Esch J, Poll LW, Hosch SB, Fritz LB, Klein M (2007). Portal vein embolization and autologous CD133+ bone marrow stem cells for liver regeneration: initial experience. Radiology.

[7] Kanazawa H, Fujimoto Y, Teratani T, Iwasaki J, Kasahara N, Negishi K (2011). Bone marrow-derived mesenchymal stem cells ameliorate hepatic ischemia reperfusion injury in a rat model. PLoS One.

[8] Dai LJ, Li HY, Guan LX, Ritchie G, Zhou JX (2009). The therapeutic potential of bone marrow-derived mesenchymal stem cells on hepatic cirrhosis. Stem Cell Res.

[9] Cho KA, Ju SY, Cho SJ, Jung YJ, Woo SY, Seoh JY (2009). Mesenchymal stem cells showed the highest potential for the regeneration of injured liver tissue compared with other subpopulations of the bone marrow. Cell Biol Int.

[10] Qihao Z, Xigu C, Guanghui C, Weiwei Z (2007). Spheroid formation and differentiation into hepatocyte-like cells of rat mesenchymal stem cell induced by co-culture with liver cells. DNA Cell Biol.

[11] Mohsin S, Shams S, Ali Nasir G, Khan M, Javaid Awan S, Khan SN (2011). Enhanced hepatic differentiation of mesenchymal stem cells after pretreatment with injured liver tissue. Differentiation.

[12] Azizi SA, Stokes D, Augelli BJ, DiGirolamo C, Prockop DJ (1998). Engraftment and migration of human bone marrow stromal cells implanted in the brains of albino rats--similarities to astrocyte grafts. Proc Natl Acad Sci U S A.

[13] Davis JM (2002). Basic cell culture.

[14] Dutta A, Bandyopadhyay S, Mandal C, Chatterjee M (2005). Development of a modified MTT assay for screening antimonial resistantfield isolates of Indian visceral leishmaniasis. Parasitol Int.

[15] Xie C, Zheng YB, Zhu HP, Peng L, Gao ZL (2009). Human bone marrow mesenchymal stem cells are resistant to HBV infection during differentiation into hepatocytes in vivo and in vitro. Cell Biol Int.

[16] Taghi GM, Ghasem Kashani Maryam H, Taghi L, Leili H, Leyla M (2012). Characterization of in vitro cultured bone marrow and adipose tissue-derived mesenchymal stem cells and their ability to express neurotrophic factors. Cell Biol Int.

[17] Stöcker E, Wullstein HK, Bräu G (1973). [Capacity of regeneration in liver epithelia of juvenile, repeated partially hepatectomized rats. Autoradiographic studies after continous infusion of 3H-thymidine (author's transl)]. Virchows Arch B Cell Pathol.

[18] Dalakas E, Newsome PN, Harrison DJ, Plevris JN (2005). Hematopoietic stem cell trafficking in liver injury. FASEB J.

[19] Miyazaki M, Akiyama I, Sakaguchi M, Nakashima E, Okada M, Kataoka K (2002). Improved conditions to induce hepatocytes from rat bone marrow cells in culture. Biochem Biophys Res Commun.

[20] Nakamura T, Nishizawa T, Hagiya M, Seki T, Shimonishi M, Sugimura A (1989). Molecular cloning and expression of human hepatocyte growth factor. Nature.

[21] Nakamura T, Teramoto H, Ichihara A (1986). Purification and characterization of a growth factor from rat platelets for mature parenchymal hepatocytes in primary cultures. Proc Natl Acad Sci USA.

[22] Gohda E, Tsubouchi H, Nakayama H, Hirono S, Sakiyama O, Takahashi K (1988). Purification and partial characterization of hepatocyte growth factor from plasma of a patient with fulminant hepatic failure. J Clin Invest.

[23] Lee KD, Kuo TK, Whang-Peng J, Chung YF, Lin CT, Chou SH (2004). In vitro hepatic differentiation of human mesenchymal stem cells. Hepatology.

[24] Baksh D, Song L, Tuan RS (2004). Adult mesenchymal stem cells: characterization, differentiation, and application in cell and gene therapy. J Cell Mol Med.

[25] Sethe S, Scutt A, Stolzing A (2006). Aging of mesenchymal stem cells. Ageing Res Rev.

[26] Pountos I, Corscadden D, Emery P, Giannoudis PV (2007). Mesenchymal stem cell tissue engineering: Techniques for isolation, expansion and application. Injury.

[27] Meirelles Lda S, Fontes AM, Covas DT, Caplan AI (2009). Mechanisms involved in the therapeutic properties of mesenchymal stem cells. Cytokine Growth Factor Rev.

[28] Baddoo M, Hill K, Wilkinson R, Gaupp D, Hughes C, Kopen GC (2003). Characterization of mesenchymal stem cells isolated from murine bone marrow by negative selection. J Cell Biochem.

[29] Chen Y, Shao JZ, Xiang LX, Dong XJ, Zhang GR (2008). Mesenchymal stem cells: A promising candidate in regenerative medicine. Int J Biochem Cell Biol.

[30] Dai LJ, Li HY, Guan LX, Ritchie G, Zhou JX (2009). The therapeutic potential of bone marrow-derived mesenchymal stem cells on hepatic cirrhosis. Stem Cell Res.

[31] Luk JM, Wang PP, Lee CK, Wang JH, Fan ST (2005). Hepatic potential of bone marrow stromal cells: Development of in vitro co-culture and intra-portal transplantation models. J Immunol Methods.

[32] Wang YJ, Liu HL, Guo HT, Wen HW, Liu J (2004). Primary hepatocyte culture in collagen gel mixture and collagen sandwich. World J Gastroenterol.

[33] Zhao R, Duncan SA (2005). Embryonic development of the liver. Hepatology.

[34] Shiojiri N, Lemire JM, Fausto N (1991). Cell lineages and oval cell progenitors in rat liver development. Cancer Res.

[35] Jakubowski A, Ambrose C, Parr M, Lincecum JM, Wang MZ, Zheng TS (2005). TWEAK induces liver progenitor cell proliferation. J Clin Invest.

[36] Gordon GJ, Coleman WB, Hixson DC, Grisham JW (2000). Liver regeneration in rats with retrosin-induced hepatocellular injury proceeds through a novel cellular response. Am J Pathol.

[37] Mall FP (1906). A study of the structural unit of the liver. Am J Anat.

[38] Rothenberg ME, Cali JJ, Sobol M, Briggs MW, Upton T (2008). Rat Hepatocyte Culture Physiology Shows Enhanced Cytochrome P450 Activity on a Synthetic Extracellular Matrix. In vitro toxicology.

[39] Lysy PA, Campard D, Smets F, Najimi M, Sokal EM (2008). Stem cells for liver tissue repair: current knowledge and perspectives. World J Gastroenterol.

[40] Fausto N (2004). Liver regeneration and repair: hepatocytes, progenitor cells, and stem cells. Hepatology.

[41] Serralta A, Donato MT, Martinez A, Pareja E, Orbis F, Castell JV (2005). Influence of preservation solution on the isolation and culture of human hepatocytes from liver grafts. Cell Transplant.

